# Supplementary material for: yQTL Pipeline: A structured computational workflow for large scale quantitative trait loci discovery and downstream visualization
Source: PLoS One. 2024 Jun 4;19(6):e0298501. doi: 10.1371/journal.pone.0298501 (PMC11149833; doi:10.1371/journal.pone.0298501)
Supplement: Attachment — Submitted filename: Review_reply_240404.pdf [file pone.0298501.s001.pdf]

We thank the Reviewers for their invaluable and constructive feedback on our manuscript. We hereby provide responses addressing each of the reviewers' comments and inquiries.

Blue: Reviewers's comments

Black: Response.

Reviewer 1:

1. Limited Customization: While the pipeline offers some level of customization, users with very specific or unconventional analysis needs may find it less flexible. For example, matrixeQTL provides more models other than linear model, e.g. ANOVA model (modelANOVA) and interaction model (modelLINEAR_CROSS).

Thank you for the suggestion, which we have now addressed. When using MatrixeQTL, the users can now specify a parameter called "model_type," which can be set to one of three values: "linear," "category," or "interaction." Selecting "linear" carries out the analysis based on *modelLinear*, which is the additive linear model provided prior to the revisions. Selecting "category" carries out the analysis based on *modelANOVA*, which treats the genotype as a categorical variable and uses an ANOVA test. Selecting "interaction" uses the *modelLINEAR_CROSS* to estimate the interactions between SNPs and a covariate. Additionally, we offer the flexibility to specify a covariate for interaction testing via another parameter, "interaction_cvrt". As in MatrixeQTL, this is by default the last covariate in the model.

2. The authors claimed that the built-in parallelization significantly reduces analysis time, as demonstrated by the case study with a ~3.5-fold speed-up. However, it's not clear how it's improved. MatrixeQTL already has a built-in parallelization mechanism to bin the data and speed it up. Does the author improve the efficiency by making more chunks (thru channels in Nextflow language)?

We apologize for the confusion. The acceleration in processing speed is achieved through Nextflow, which can execute multiple processes in parallel.

While MatrixeQTL does offer efficient built-in parallelization, in theory without an upper limit on the size of the data input, the memory requirement for the matrix multiplication increases significantly as the size of genotype, phenotype, and sample data increases. The memory requirements can thus exceed practical limits for large datasets and splitting the input data into smaller data subsets ("chunks") becomes necessary.

For instance, in our own data analysis, we had a GDS file containing 9 million SNPs and a sample size of 194. On a large memory machine equipped with 32 GB of memory, we had to restrict the number of phenotypes (i.e., metabolites) included in a single run to 200 to prevent crashing. Although 200 phenotypes may initially appear ample, in practical QTL discovery analyses, researchers often work with thousands of phenotypes, for example, gene or protein expression data. Additionally, researchers may be dealing with a substantial number of samples and potentially an even greater number of SNPs to

analyze. As a solution, we developed the pipeline capable of splitting processes and executing multiple MatrixeQTL engines in parallel.

A clarification of this point has been added to the discussion section (line 333 to 337).

3. For non-familial data, the pipeline uses matrixeQTL as its engine, "to take advantage of MatrixeQTL's greater efficiency". However, there are tools such as fastqtl which are known to have better efficiency than MatrixeQTL. The author should consider it and even better make it optional allowing users to choose their own engine.

We appreciate the suggestion and recognize that other tools exist. We have chosen the tools we included because of their use of similar input data format (e.g., GDS format) and language implementation (R), which makes their integration more efficient and minimizes data re-processing. While fastqtl presents promise as a tool, its input requirements differ from those of MatrixeQTL and GENESIS. Integrating fastqtl into the workflow via Nextflow would introduce complexity, particularly concerning file input and output formats dependent on the engine in use. It thus goes beyond the current scope of our proposed tool, although we may consider it for future implementations.

4. Data visualization: Other than the Manhattan plot, the tool should also generate box plots for the significant phenotype-genotype pairs, which are commonly used in xQTL publications. Also, instead of Manhattan plot, the author could consider using Miami plot by splitting the QTL results with +/- coefficients.

We greatly appreciate the reviewer's suggestion. We have implemented the Miami plot in the R Shiny app alongside the Manhattan plot. Furthermore, we now have the pipeline automatically generate genotype-phenotype boxplots for the top SNP during the analysis step. Additionally, in the Shiny App, users now have the option to upload genotype and phenotype files and draw genotype-phenotype boxplots for a user-selected SNP. We sincerely thank the reviewer for their contribution to the improvement of our tool.

Below are the screenshots from the Shiny App:

Manhattan plot:

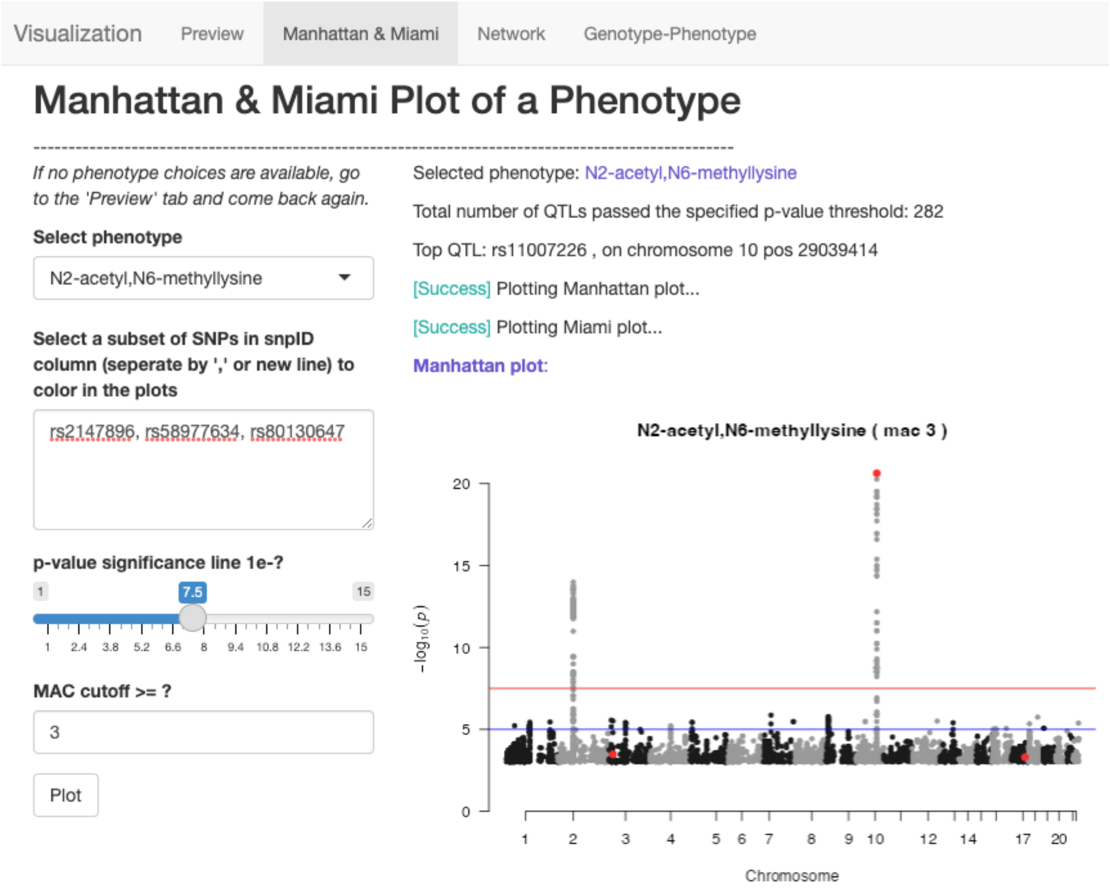

Scroll down to see the Miami plot:

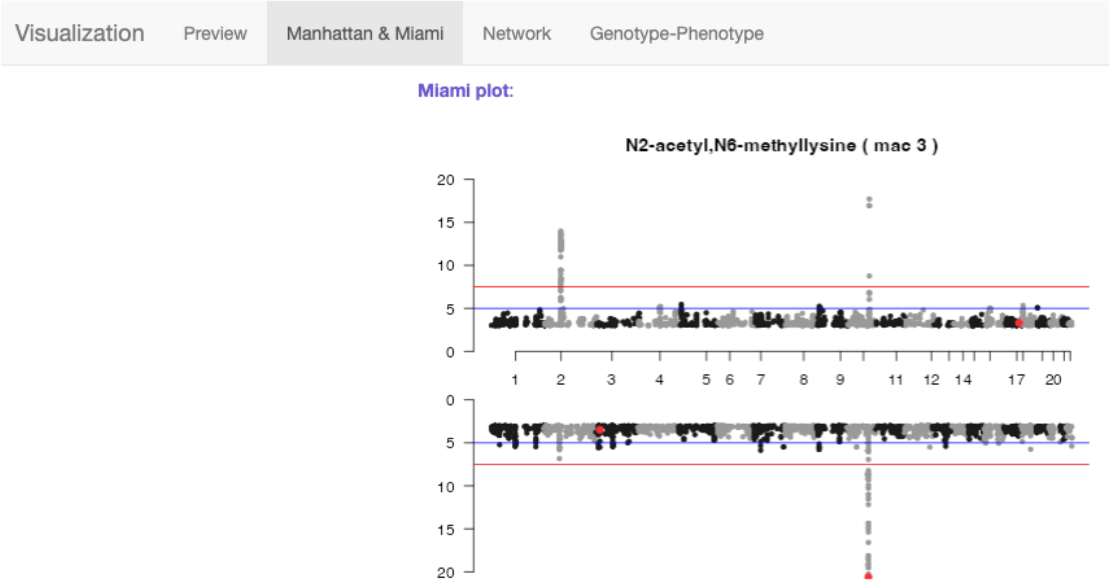

Genotype-phenotype boxplot:

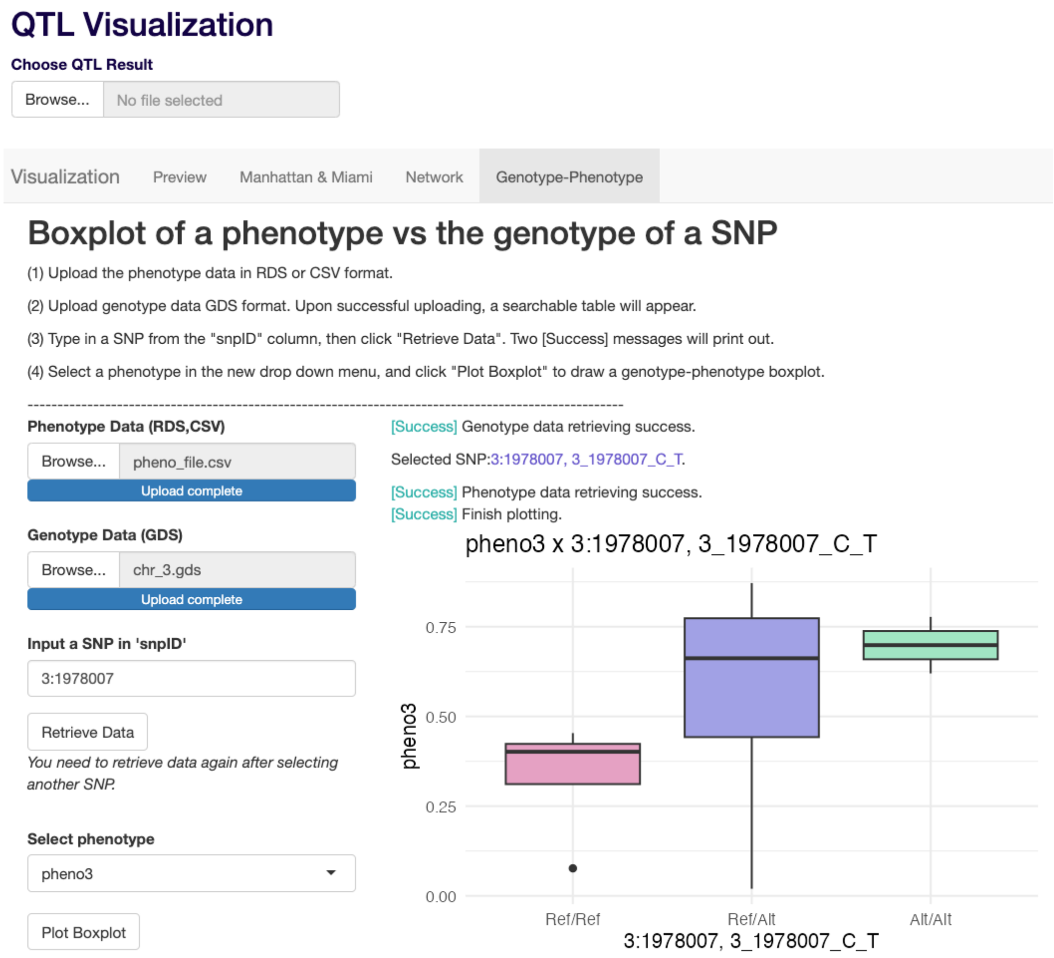

5. Data visualization again: Unlike GWAS result (which is SNP centered), QTL result is for phenotype-genotype pair. So, the author could consider generating two kinds of locus plots, one viewed as the query SNP (where each dot is a gene in the cis window), and one viewed as the query gene (where each dot is a SNP in the cis window).

This is a very interesting suggestion. However, upon closer examination, we have decided against its implementation and incorporation in the interface.

We illustrate our reasoning below, using results from a protein-QTL (pQTL) analysis of 4,397 protein aptamer expression levels and 194 samples, since the example used in the paper pertains to metabolite data, which do not possess specific "loci" on the genome. We only kept results that passed the p<1e-3 threshold to remove noise.

The first limitation is that most of the SNPs are only associated with a very limited set of phenotypes, as per the example below. The plot is showing all associations passing the p<1e-3 threshold for a SNP, and each dot is a protein. The x-axis is the starting point of the corresponding protein-coding genes. In this case, this “Manhattan plot” provides little additional information compared to a network plot where the researcher can clearly see the SNP’s connection to several proteins.

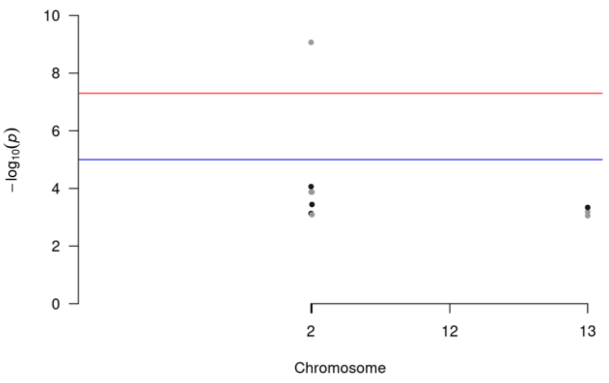

In rare cases, a SNP can be found to be significantly associated with a relatively large number of phenotypes. For example, in our pQTL analysis, there were 20 SNPs associated with more than 400 proteins at p < 1e-3. Below, we show one such case (with each dot representing a protein, and with its x-axis coordinate reflecting its genomic starting location). Even in this instance, the Manhattan plot does not provide much additional value. The reason is, in GWAS analysis, we examine Manhattan plots primarily to identify "signals", i.e. “skyscrapers,” as SNPs tend to form “clusters” with their neighbor since they are in high LD with each other. However, this phenomenon is not applicable on the phenotype side. Multiple proteins may share the same QTL, but they are not in “LD” with their neighbor proteins on the genome.

As illustrated in the example below, the signals are distributed across the entire genome, and the presence or absence of a "skyscraper" does not validate nor invalidate the signals. In cases where users wish to examine which proteins are significantly associated with a particular SNP, a network plot would be still more insightful.

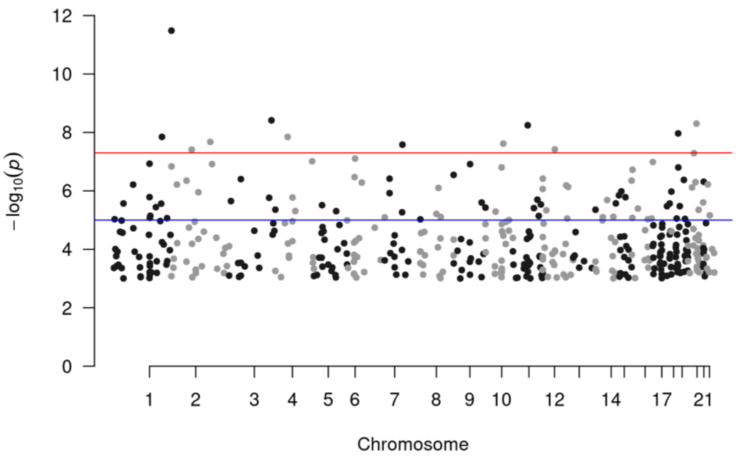

Finally, it's important to note that the "phenotype-based Manhattan plot" is only applicable to a limited set of phenotypes, such as gene expression, protein expression, and methylation sites. Other quantitative phenotypes like metabolite expression, testing scores, or medical measurements do not possess "loci" on the genome, and therefore are not suitable.

6. This might be beyond the scope of the work, but since the author developed the yQTL Pipeline to incorporate all the analysis steps into a single pipeline, it's worthy to include pre-QC steps into the pipeline. For example, early work like https://bmcbioinformatics.biomedcentral.com/articles/10.1186/s12859-021-04307-0 has well characterized the necessary step for input data QC for QTL analysis.

We greatly appreciate the suggestion. As a response, we have developed PreQC.nf, which operates independently of the other steps, serving as an "add-on" feature for users to perform as needed. This script conducts pre-quality control on the input genotype data. It has the capability to filter out SNPs based on Hardy-Weinberg equilibrium (HWE) violation, missingness, and minor allele count. Users have the flexibility to define the HWE p-value threshold to identify SNPs violating HWE, as well as the thresholds for missingness and minor allele count.

7. the link https://github.com/montilab/yQTLpipeline does not work at the moment of review.

We deeply apologize. The link above is now working.

Reviewer 2

1. The GitHub page mentioned for accessing the pipeline appears to be unavailable or not correctly linked (https://github.com/montilab/yQTLpipeline). Could you provide the correct URL?

We deeply apologize. The link above is now working.

2. It would be beneficial to know if the pipeline's runtime has been compared with that of other tools such as GENESIS and/or MatrixeQTL. Can you share any comparative analysis results?

Please also see response to Reviewer 1's question 2.

The actual runtime difference may vary depending on the specific case. As demonstrated in the example provided in the paper, conducting our metabolite QTL analysis by manually running MatrixeQTL step-by-step would require approximately 90 minutes. While MatrixeQTL in theory does not set limits on the number of phenotypes to be analyzed simultaneously, the memory requirement may exceed a machine capacity for large datasets. When running on a 32 GB memory machine, we had to split the phenotype set into groups of 200 phenotypes each to prevent crashing. Therefore, the 90-minute timeframe is from the cumulative time of running MatrixeQTL multiple times for this QTL discovery task. However, utilizing our pipeline reduces the time required for the user to 26 minutes, as processes are executed in parallel across multiple machines whenever feasible.

When applied to larger datasets, such as those containing thousands of samples and tens of millions of SNPs, the execution time reduction has the potential of becoming even more pronounced thanks to parallelization (assuming availability of multiple nodes).

3. Is the pipeline equipped to perform conditional eQTL analysis? If so, could you elaborate on this functionality?

We appreciate the suggestion, but we feel that the implementation of conditional yQTL analysis goes beyond the scope of this pipeline, as it is very case-specific, and its general purpose implementation would drastically increase the complexity of the workflow.

In conditional yQTL, selection of the loci warranting conditional analysis usually requires the analyst's judgement, and possibly manual selection of one specific locus. Subsequent conditioning on the top SNP and rerunning of the analysis is followed by the need for the analyst to reassess the necessity of further conditioning on the current top SNP, and to perform additional analyses. While this iterative process is conceptually straightforward, its automatization is not trivial, and we believe goes beyond the scope of a pipeline meant for large-scale screening.

4. Does the pipeline incorporate any genotype quality control (QC) or SNP filtering steps? Clarification on this would be helpful.

Thank you for the suggestion. See response to Reviewer 1's question 6.

5. Regarding the visualization features, is it possible to specifically highlight QTLs of interest for presentation purposes?

We greatly appreciate the suggestion. We have implemented this feature in our R Shiny App, enabling users to specify a set of SNPs to be highlighted when drawing the Manhattan plot.

Please see the screen shot in the response to Reviewer 1's question 4. The red dots in the Manhattan plots are the user selected SNPs.
